# “Hormones rule me”: A qualitative exploration of the impact of perimenopause on women with ADHD

**DOI:** 10.1177/17455057261450178

**Published:** 2026-07-17

**Authors:** Christina Kini-Seery, Samantha Trevaskis, Ken Kilbride, Margo Wrigley, Jessica Bramham

**Affiliations:** 1 320791 UCD School of Psychology, University College Dublin, Dublin, Ireland; 2 ADHD Ireland, Carmichael Centre for Non-Profits, Dublin, Ireland; 3 National Clinical Programme for ADHD in Adults, 8004 Health Service Executive, Dr Steeven’s Hospital, Dublin 8, Ireland

**Keywords:** ADHD, attention deficit hyperactivity disorder, perimenopause, menopause, women’s health, premenstrual dysphoric disorder

## Abstract

**Background:**

Perimenopause appears to be a particularly complicated period for women with Attention-Deficit Hyperactivity Disorder (ADHD), who report increased physical, sexual, and mood-related symptoms compared to neurotypical women.

**Objectives:**

The present study sought to investigate qualitatively how women with ADHD experience perimenopause.

**Design:**

The present study employed a qualitative, phenomenological design.

**Methods:**

Nineteen cisgender women with ADHD residing in Ireland (age range = 29-62, *M* = 47.17, *SD* = 6.59) participated in semi-structured. Interviews were conducted online, via Zoom or email, and transcribed verbatim. Data were analysed using Reflexive Thematic Analysis.

**Results:**

Four themes were developed. *“It literally just hit me like a punch in the face”: Navigating emotional and cognitive changes* showcased the impact on ADHD and mental health. *“I ache and I feel old”: Navigating physical changes* explored how the physical symptoms of perimenopause affect women relationship to their bodies, especially sexual symptoms. *“Whatever I’m searching for perimenopause, it should be within the light of ADHD”: Navigating healthcare* highlighted the importance of integrated care for ADHD and perimenopause. *“It’s like going through a fire that burns off all that you don’t need anymore”: Finding the positives* suggested that while challenging, perimenopause could bring new compassion and understanding for ADHD.

**Conclusion:**

These results highlight the complications and difficulties experienced by women with ADHD during perimenopause, and the importance of supporting them with integrated, affirming healthcare.

## Introduction

ADHD is a neurotype characterised by differences in attention regulation, hyperactivity, and impulsivity.^1^ The estimated prevalence of adult ADHD is 2.5-3% globally.^2,3^ Men are more likely to have an ADHD diagnosis than women, with a prevalence of 4.3% vs 3%,^3^ likely due to factors such as diagnostic criteria, overshadowing, and sociocultural expectations.^4^ A cohort study of individuals with ADHD identified that women with ADHD were more likely to receive a mental health diagnosis, such as depression or anxiety, prior to their ADHD being recognised than men with ADHD, suggesting increased rates of misdiagnosis or diagnostic overshadowing.^5^ Women with ADHD demonstrate an increased vulnerability to mental health difficulties.^6–9^ Given the significant mental health problems many girls and women with ADHD experience, it is vital to understand the causal and maintaining mechanisms of psychological distress across their lifespan to develop targeted interventions.^10^ One such mechanism appears to be hormonal transitions.

There appears to be an important and complicated relationship between ADHD and female sex hormones, with studies suggesting increased emotion dysregulation, executive functioning and concentration difficulties in girls and women during their luteal phase.^11^ Recent small pilot research has identified that stimulants for ADHD may be less effective during the luteal phase.^12^ Additionally, women with ADHD have a higher prevalence of premenstrual dysphoric disorder and episodes of postpartum depression than neurotypical women,^13^ as well as being more vulnerable to depression when using hormonal contraception.^8^ During periods of hormonal change, women with ADHD experience a threefold increase in the severity and frequency of mood symptoms.^14^ Given these difficulties, it is important to explore the lived experience of periods of hormonal changes for women with ADHD to inform clinical practice.

Perimenopause, or the transition phase prior to the menstrual cycle ceasing for at least one year, is marked by fluctuating imbalances in oestrogen and progesterone hormones.^15^ The relationship between ADHD and perimenopause is an emerging area of interest. It has been proposed that individuals with ADHD have increased sensitivity to hormonal changes as periods of reduced oestrogen circulation are associated with decreased dopamine production, causing increased executive functioning difficulties,^16^ as research has demonstrated increased ADHD traits when oestrogen is lowered.^17^ Additionally, perimenopause is marked by cognitive changes, such as decreased processing speed, attention, and working memory,^18^ which may be especially challenging for women with ADHD. A large-scale survey of readers of ADDitude magazine, an ADHD-focused publication, found that a majority of participants (61%) reported that ADHD had the greatest impact on their lives during typical perimenopause years. Cognitive difficulties reportedly peaked during perimenopause.^19^ L. Chapman et al. observed significantly reduced quality of life in relation to sleep, memory and concentration in women with ADHD compared to neurotypical women, but no differences in perimenopause symptoms. However, they found a significant positive association between perimenopause symptoms and ADHD traits.^20^ Boyd demonstrated significantly higher levels of anxiety, depression, and physical and sexual dysfunction symptoms in women with ADHD than in neurotypical women.^21^ Similarly, a higher prevalence of severe perimenopausal symptoms, including psychological, somatic, and urogenital, was observed in women with ADHD than in those without and perimenopause symptom-onset was at an earlier age for women with ADHD.^22^ Given that women with ADHD appear to experience perimenopausal symptoms to a greater degree, it is helpful to explore how women with ADHD experience and conceptualise these increased symptoms of perimenopause and the impact they have on their lives.

While there is new evidence to show elevated difficulties during menopause for women with ADHD, no studies to our knowledge have yet qualitatively investigated this phenomenon. In comparison, some research has explored the experience of menopause in autistic women. A focus group with autistic individuals found that not only were their pre-existing difficulties exacerbated during perimenopause, but they also experienced additional cognitive, social, and emotional challenges that significantly impacted their mental health.^23^ Another interview study with autistic people found that during perimenopause, there was a reported increase in suicidal feelings and self-harm behaviours.^24^ Similarly, Brady et al. utilised qualitative research to identify several barriers to support and care in relation to navigating healthcare for perimenopause as an autistic individual.^25^ Qualitative research could allow researchers and clinicians to understand the specific needs and challenges of women with ADHD during perimenopause.

The present study aimed to qualitatively explore how women with ADHD experience perimenopause. We were interested in identifying lived experience perspectives on how perimenopause affects ADHD traits and mental health, and what, if anything, supports women with ADHD to cope during the transition to menopause.

## Methods

The present study employed a qualitative, phenomenological design using semi-structured interviews. Ethical approval was granted by the first and senior authors’ host institution (HS-25-38-Seery-Bramham). All participants provided informed consent. Participants were included if they were over 18, either currently experiencing perimenopause symptoms or have transitioned through menopause, fluent in English, resided in Ireland or the United Kingdom, reported a formal diagnosis or self-identified ADHD, and met clinical levels of ADHD traits measured by the ADHD Self-Report Scale.^26^ No exclusion criterion based on the type of menopause (natural, chemical, or surgical) was applied, as recommended by the lived experience expert and in line with previous research on autistic experiences of perimenopause.^27^ The reporting of this study conforms to the COREQ statement,^28^ which is available in the Online Supplementary Materials.

## Materials

***ADHD Self-Report Scale (ASRS)***.^26^ The ASRS includes 18 questions about the frequency of DSM-IV traits of adult ADHD. Participants rate their answers on a scale of ‘never’ to ‘very often’. The first six items are indicative of the presence of ADHD, with the remaining twelve items providing further context for traits. A total score of 14 or higher indicates clinical levels of ADHD traits.^29^

***Interview schedule***. The interview schedule was developed drawing on previous qualitative research conducted with autistic individuals on their experience of the menopause transition.^23,25^ Questions were adapted to reflect the ADHD experience, with additional topics added based on a review of the literature and following extensive discussion between the first and senior author. A draft of the interview schedule was shared with a researcher specialising in women’s health, who has lived experience of ADHD and menopause, for feedback and further development. The interview schedule is provided in Table 1.

**Table 1.** Interview schedule.

Question	Prompts if necessary or supportive
What has been your experience of perimenopause or menopause overall?	Did you recognise your symptoms as perimenopause? What was your understanding of menopause when your symptoms started? Did realising you were experiencing the menopause transition affect how you viewed yourself? Did you know you had ADHD when your symptoms started? How did you experience menstruation before perimenopause/menopause? How, if at all, do you think menopause affects your experience of your ADHD? Do you think people with ADHD experience menopause differently to neurotypical people?
What do you struggle with on a day-to-day basis? How has this changed since perimenopause?	​
Executive functioning is the brain’s self-management system, responsible for things like organising, focusing, impulsivity, hyperactivity, memory, and processing speed. How was perimenopause or menopause impacted on your executive functioning?	How, if at all, do you think your traits of ADHD have changed since perimenopause? How has working/work-life been for you? Do you or have you ever masked, hid, or camouflaged your ADHD traits? Have your experiences of menopause interacted with masking/camouflaging?
If you think about what your experience of your mood or mental health was before your symptoms, what has been your experience of your mental health and mood since perimenopause?	How, if at all, has your mental health changed? What has the impact of your mental health been on your daily life? How have you experienced your emotion regulation since perimenopause? Did you notice changes in your emotion regulation abilities?
What has been your experience of the physical symptoms of perimenopause or menopause?	How have you experienced the physical symptoms of menopause (e.g., hot flushes, headaches, insomnia, vaginal dryness, UTIs, hair thinning)? How have these impacted on your daily life? What has been your experience of sensory sensitivities (e.g., sensitivity to sound, light, smells, food textures, tastes). Have these changed since perimenopause? What has that been like? What has been your experience of your sexuality and sexual intimacy since perimenopause? How has this impacted you?
What supports have you utilised or sought?	Have you sought medical support or treatment, like Hormone Replacement Therapy? What has that experience been like? Do you take ADHD medication? What has been your experience of ADHD medication since perimenopause if so? Have you visited a medical or healthcare professional (e.g., GP or specialist menopause clinic) for support? How was that experience? Do you know if they were a specialist in menopause? What do you wish you would have known about menopause and ADHD? What information do you get about menopause and where do you get it form? What tools have you implemented yourself to cope or thrive during perimenopause or menopause (e.g., exercise, sleep, nutrition)?
Overall, how do you think menopause has impacted on your quality of life?	​
Is there anything else you would like to add that we didn’t get to discuss?	​

### Procedure

Data were collected with the aim of integrating neuro-affirmative principles^30^ throughout the procedure. Interviews were conducted June to August 2025. Convenience sampling was using as the research was advertised on the first author’s own social media and promoted by ADHD Ireland, Ireland’s national charity for ADHD, through their website, mailing list and social media platforms on LinkedIn, X, and Instagram. Advertisements included an overview of rationale for research (such as research suggesting women with ADHD experience more complications during perimenopause), inclusion criteria, study methodology, and how to find out more information. The rationale for the research may have prompted women with more challenges during perimenopause to express interest in participating. Individuals interested in participating contacted the first author to confirm their eligibility.

To determine the necessary sample size of the study, the research team followed Braun and Clarke’s recommendations to employ pragmatic considerations rather than aiming for data saturation.^31^ They argue that data saturation is incongruent with Reflexive Thematic Analysis, the data analysis approach adopted for the present study, as the meaningfulness of data is not tied into the number of datapoints but the richness of analysis. Instead, the research team considered discipline norms of psychology, the time and financial constraints of the project, and interviewer capacity.^31^ As such, a target of 15-20 interviews was set, with participants recruited to allow for attrition. Of the individuals who expressed interest in the study, CKS contacted the first 37 individuals who emailed her to provide them with further information and invite them to participate. Fifteen participants did not respond after their initial email and three cancelled their interviews due to personal circumstances. The final sample consisted of nineteen participants.

Given the increasing challenge of ‘imposter participants’ in qualitative studies,^32^ CKS followed Lough Dennell et al.’s approach of emphasising human connection in the interviews, such as getting to know the participant before the interview is recorded, considering their motivations and sense of urgency in expressions of interest in participating, and by the details provided in responses to interview questions.^33^ All participants seemed to be genuine and motivated to support research in the area.

All participants provided informed consent to participate by providing an online signature to the consent sheet following the information sheet. Interviews were facilitated by the first author, CKS. Participants did not have a prior relationship with CKS before the study. The information sheet informed participants of the nature of CKS’s role (postdoctoral researcher working in the area of hormonal transitions for individuals with ADHD) and her relationship to the study. Adults with ADHD experience a range of executive functioning differences, including differences in their organisation, focus, impulsivity, emotion regulation, working memory, and processing speed.^34^ These differences can be exacerbated during perimenopause.^35^ Several accommodations were implemented to support participants’ executive functioning during the interviews, to allow them to fully explore their experiences while adopting a neuro-affirming approach. Participants were provided with the interview schedule in advance to reduce participatory anxiety and support processing speed differences. Participants were also invited to participate in an online, recorded interview (*n* = 16) or an asynchronous, email-based interview (*n* = 3). Flexible data collection measures were facilitated for participants who were limited in terms of time or for those who had memory or processing speed differences. Participants in the asynchronous interviews replied to the questions via email, with the first author sending additional prompting or clarifying questions. The first author facilitated the synchronous interviews. The interviews adopted a conversational style, emphasising participants’ comfort and safety over strict adherence to the interview schedule. Prompts were provided when participants benefitted from additional directions following open-ended questions (see recommendations from Moseley et al.^35^). Only the interviewer and participant were present during the interviews. Following completion of the interview, the author noted thoughts and reflections that arose for her. The length of synchronous interviews ranged from 24.12 to 60 minutes (*M* = 37.54, *SD* = 11.62).

### Participants

Nineteen cisgender women with ADHD participated in the study. To meet inclusion criteria, participants identified as perimenopausal or post-menopausal. Menopause is classified as not having a period for at least 12 months.^36^ Using this definition, five participants were post-menopausal. The remaining 74% of participants reported ongoing irregular menstruation and were currently perimenopausal. Excluding a participant who recently underwent chemical menopause (initiated three months before the interview), the mean length of time participants reported experiencing perimenopause symptoms was 5.26 years (*SD* = 2.90, range = 1-10 years).

Thirteen participants reported that they received a formal diagnosis of ADHD, and six participants self-identified as having ADHD. All participants met the ASRS cut-off score for clinically significant levels of ADHD (*M* = 23.94, *SD* = 2.80, range = 19-27). The majority of those who were formally diagnosed were diagnosed since perimenopause symptoms began (*n* = 10; 77%). The mean age was 47.17 (*SD* = 6.59, range = 29-62). Additional participant demographics are provided in Table 2.

**Table 2.** Participant demographics and characteristics.

Demographic characteristic	N
**Employment status**	
Employed full-time	13 (68%)
Employed part-time	2 (11%)
Retired	1 (5%)
Unemployed, unrelated to retirement	3 (16%)
**Race**	
White Irish	13 (68%)
White Other	5 (27%)
Latina	1 (5%)
**Sexuality**	
Heterosexual	18 (95%)
Bisexual	1 (5%)
**Menopause Means**	
Natural means	16 (84%)
Chemical intervention	3 (16%)
**History of Gynaecological Disorders**	
Yes	11 (58%)
No	8 (42%)
**Types of Gynaecological Disorders** *	
Endometriosis	4 (36%)
Fibroids	4 (36%)
Dysmenorrhea	2 (18%)
Polyps	2 (18%)
Ovarian Cysts	2 (18%)

* = Several participants reported history of multiple gynaecological disorders.

### Data analysis and reflexivity

**Figure 1.**
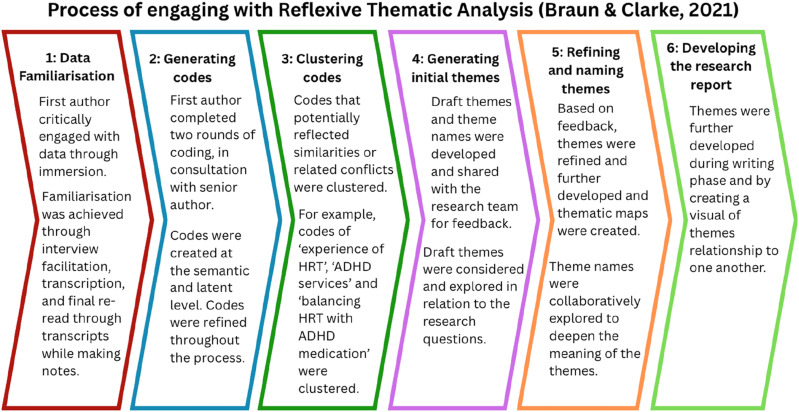
Process of engaging with the six key stages of reflexive thematic analysis

Data were transcribed verbatim. Reflexive thematic analysis (RTA)^37^ with an ontological approach of critical realism^38,39^ was employed to analyse the data. The phases of RTA utilised are reported in Figure 1. A predominantly inductive approach was applied. NVivo 20^40^ was used for the coding process.

The research team consisted of academic, clinical, and lived experience expertise. The first author, CKS, is a postdoctoral researcher with a PhD in Psychology, with research interests in clinical psychology and neurodiversity. She has completed advanced training in qualitative methods and analysis, and previously facilitated semi-structured and narrative interviews with individuals from marginalised groups. While she is neurodivergent, she does not have lived experience of ADHD or perimenopause, and was therefore an ‘outsider’ to participants’ experiences. Alongside her postdoctoral research, she facilitates groups for adults with ADHD that provide psychoeducation in combination with Acceptance and Commitment Therapy^41,42^ and as such, her clinical experience of working with women with ADHD in perimenopause attending the groups informed her engagement with the research. ST is a women’s health researcher with lived experience of ADHD and menopause. KK is the CEO of ADHD Ireland, and MW is the Clinical Lead for the National Clinical Programme for ADHD in Adults in Ireland. JB is an academic neuropsychologist, specialising in adults with ADHD. All of the research team provided feedback and input on theme development to CKS.

With awareness of the societal context (see supplementary materials for details on the Irish context in relation to menopause and ADHD) as well as her own influences on the research, the first author maintained a reflexivity journal, noting her own observations and assumptions throughout all stages of the research. To support reflexivity, the first author consulted with ST on the theme development, which helped to enhance her understanding and facilitated further exploration with this additional perspective. Additionally, the first author discussed assumptions, observations, and potential influences in depth with the senior author as they arose throughout the study.

Participant quotes are presented with a pseudonym (of their choosing unless they requested the researcher choose one for them) and their age.

## Results

**Figure 2.**
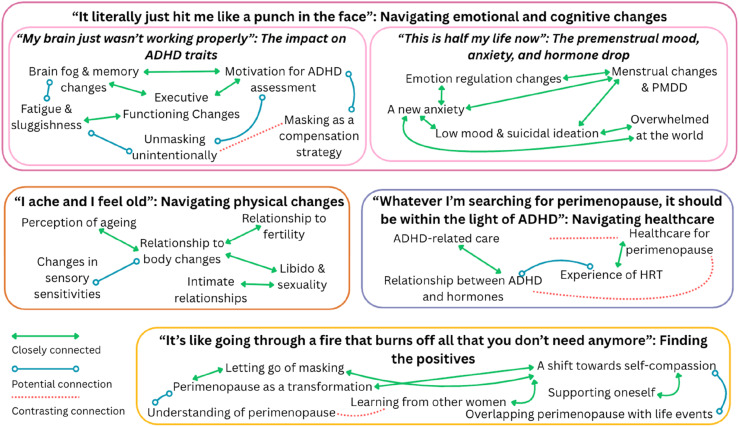
Thematic map demonstrating codes’ relationships (closely connected, potential connection, contrasting connection) to one another.

**Figure 3.**
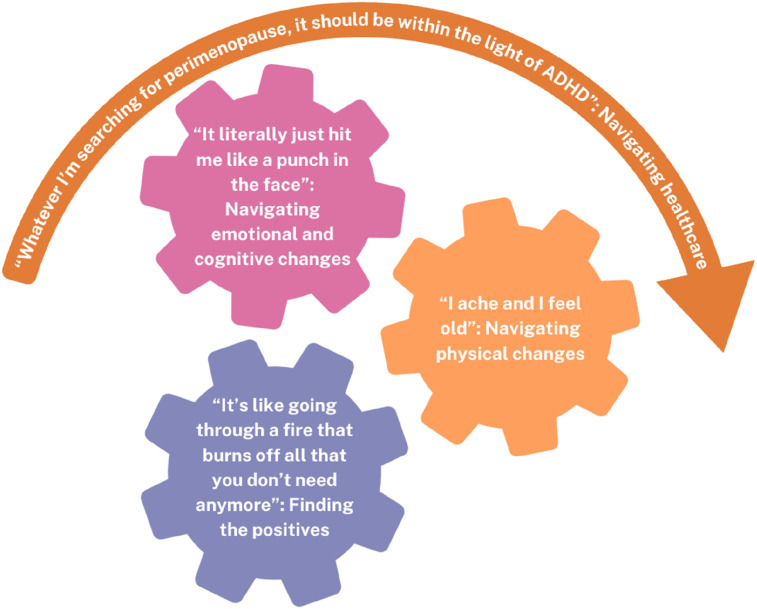
A proposed model of the themes’ relationship to one another. *Note.* This figure depicts three of the themes as cogs to reflect how they influence each other – how emotional, cognitive, and physical changes are navigated affects the way individuals were able to positively adapt to perimenopause. The theme on navigating healthcare is depicted as an arrow to highlight that this is a mechanism underpinning the remaining three themes.

RTA generated four themes. An overview of the codes associated with each theme is presented in Table 3. Figure 2 provides a thematic map and identifies how codes relate to one another within a theme, while Figure 3 provides a visual model of how each of the themes influences one another. Overall, the themes highlight the profound impact of hormonal changes and perimenopause for women with ADHD, as highlighted by Rachel (49): *“Life changing, yeah, yeah, I tell you, I didn’t realise obviously, how big a deal it would be. I think hormones like rule me. I think I’m just very sensitive to the changes.”*

**Table 3.** Framework of codes applied to themes.

Theme	Related codes
“It literally just hit me like a punch in the face”: Navigating emotional and cognitive changes	*“My brain just wasn’t working properly”: The impact on ADHD traits* subtheme Brain fog & memory changes; Executive functioning changes; Fatigue & sluggishness; Unmasking Unintentionally; Masking as a compensation strategy; Motivation for ADHD assessment *“This is half my life now”: The premenstrual mood, anxiety, and hormone drop* subtheme Menstrual changes & PMDD; Emotion regulation changes; A new anxiety; Low mood & suicidal ideation; Overwhelmed at the world
“I ache and I feel old”: Navigating physical changes	Relationship to body changes; Perception of ageing; Relationship to fertility; Changes in sensory sensitivities; Libido & sexuality; Intimate relationships
“Whatever I’m searching for perimenopause, it should be within the light of ADHD”: Navigating healthcare	Healthcare for perimenopause; Experience of HRT; ADHD-related care & services; Relationship between ADHD and hormones
“It’s like going through a fire that burns off all that you don’t need anymore”: Finding the positives	A shift towards self-compassion; Letting go of masking; Perimenopause as a transformation; Understanding of perimenopause; Supporting oneself; Overlapping perimenopause with life events; Learning from other women

### “It literally just hit me like a punch in the face”: Navigating emotional and cognitive changes

The first theme captures the reported changes to the participants’ inner worlds and highlights the shame and challenges associated with them. Two subthemes were developed to capture these experiences. Many of the women experienced these emotional and cognitive changes as sudden, forceful, and disruptive to their lives: *“It [a hormonal mood drop] literally just hit me like a punch in the face, and I had no idea if it was going to go away. And I was in complete despair”* (Aileen, 46).

#### “My brain just wasn’t working properly”: The impact on ADHD traits

All participants reported that perimenopause exacerbated their ADHD executive functioning differences. There was frequently a sense of losing one’s mind, the brain being out of control, or that it was broken, as demonstrated by Katie’s (39) quote: *“My brain just wasn’t working properly”*. The most challenging change was declining memory and brain fog. While working memory had typically been a difference they experienced throughout their lives, it became significantly worse:

“I read a couple of articles about women with ADHD who think that they have onset of dementia. And I very much felt that, because, like I would have been, you know, when I was younger, I was away with fairies and whatever, but I never noticed it. But Jesus Christ, when I started going through Perimenopause. … [it] just felt like a little bit scary, you know” (Louise, 52).

Other changes to executive functioning noted were word finding, fatigue and sluggishness, and increased distractedness, hyperactivity, and restlessness. Some women reported a sense of being drained and exhausted, and the impact that has on their work life by needing more opportunities to rest: *“I am self-employed/freelance... Sometimes because jobs overlap, I need longer and longer breaks between looking for work to recover, yet I never seem to regain my energy”* (Caroline, 58). The increased hyperactivity and restlessness were described as both an internal hyperactivity or buzzy brain and physically needing to move around more, and a sense of being energetically wired constantly:

“I was absolutely hyper in my brain. It feels like, the only way I can describe it is like a pinball machine, but there's lots of balls going around. It's not just one ball. There's lots. ... I would find it very difficult, and yeah, probably again as I hit my 40s, to rest, to allow myself to rest, to be able to sit, like I can't sit down.” (Beyonce, 46).

Participants reported several consequences of the changes in their ADHD traits. Frustration and embarrassment were frequently described, particularly as executive functioning was impacted at work. They could also have an impact on other people, especially family members, which led to feelings of guilt and shame:

“My son particularly, has a really hard time because he wants to tell me something, and I'm just, you know, and I cut him off because I want to move on to a different topic already. And so that does not work. It results in tears. It's super hard.” (Rose, 46).

Changes in executive functioning also disrupted the ability to mask ADHD traits. Participants shared that other compensation strategies for ADHD were no longer sustainable: *“Considering I now know I had ADHD all along, I was using ways to manage it without realising it, and when perimenopause started to kick in, those coping mechanisms weren’t working any more”* (Miriam, 48). For participants who were diagnosed since perimenopause, ADHD traits flaring to a difficult degree and being unable to respond with previous coping mechanisms were often the catalyst to seeking an assessment.

#### “This is half my life now”: The premenstrual mood, anxiety, and hormonal drop

Alongside changes to cognition, almost all participants reported significant differences in emotion and mood regulation, which were especially evident during the luteal phase of their cycles. Several participants had been diagnosed with Premenstrual Dysphoric Disorder (PMDD). Those with a PMDD diagnosis felt it worsened during perimenopause. For many women, their hormonal changes during the luteal phase led to an overwhelming sense that half their month, or *“this is half my life now”* (Katie, 39), would be marked by anxiety and depressive symptoms and emotional dysregulation. Betty (48) compared her follicular versus luteal phase to the story of Dr Jekyll and Mr Hyde, a character struggling between dual personalities.

Hormonal mood drops represented a significant impairment in quality of life during the luteal phase, including guilt regarding the impact of mood changes on family members. Even with Hormonal Replacement Therapy, participants noticed their mood was affected by their luteal phase. An important way of coping is to recognise the temporary nature of the change in mood, note what day during the cycle it is, and respond by self-isolating:

“I’ll be having a bad day, and I’ll be like, f**k sake, oh, this is it. I mean, I don’t I don’t want to be married anymore, and I don’t want to do this job, and blah, blah, blah, and then I’ll hear the other voice again. *Day 14. Day 14. Do not talk to many people today*” (Rachel, 49).

Participants identified that the anxiety symptoms they felt during perimenopause was distinct from the anxiety they had experienced throughout their lives. It manifested as a strong sense of bracing for the worst-case scenario. What distinguished it from previous anxiety was the degree to which symptoms felt physical and embodied: *“I was having heart palpitations, panic attacks and sweating… just basically out of whack”* (Claire, 44). When anxiety was being experienced, it could grow or *“mushroom”* (Louise, 52) into something uncontrollable. Participants related the anxiety symptoms to feeling overwhelmed with the world and a sense of being chronically unable to manage.

Feeling overwhelmed was also associated with low mood and depressive symptoms that were reported. For those who experienced depressive symptoms, most felt it as an acute, sharp drop in mood that caught them off guard:

“I went from being, you know, I'm fine, pottering away, doing my thing to *I would just toss myself in that river*, and I'm done, you know, it just dropped so fast. And it literally felt like, you know, if somebody was to test my hormones and say, your hormones dropped, that's the only thing I can think, like, after you have a baby, I have this, like, hormone sink too” (Aileen, 46).

As demonstrated by Aileen, several participants reported suicidal ideation as a result of the hormonal drop in mood. Suicidal ideation ranged from a wish to cease existence, *“I’m not suicidal, but I'm telling you, if there was a big white button on that wall that would make everything stop, I’d be hitting it, you know”* (Rachel, 49), to researching the impact of dying by suicide on loved ones. This highlights the seriousness and significance of the hormonal mood drops experienced more often during the luteal phase.

Overall, these two subthemes reflect the significant impact of cognitive and emotional changes during perimenopause for women with ADHD, and the importance of providing support to minimise the effects on their quality of life and risk of suicide.

### “I ache and I feel old”: Navigating physical changes

The physical symptoms of perimenopause were experienced as challenging in several aspects. Most common symptoms reported were joint aches, weight gain, sweating, hot flashes, hair loss, vaginal dryness and in some cases, vaginal atrophy: *“I ache and I feel old, and I can’t move, and I’m not me, and my weight, you know, skyrocketed, and then they’re like, maybe you’re depressed about your weight”* (Rachel, 49). Physical symptoms felt disjointed until hot flashes started, which was typically what prompted participants to realise they were in perimenopause.

Physical changes often led to self-esteem and the individual’s relationship to the body hitting *“absolute rock bottom”* (Maisie, 46). For some, the changes represented a loss of identity. Physical symptoms, weight gain in particular, meant bodily changes felt out of control, no matter what the individual tried to do to address them, and resulted in a sense of disconnect:

“I feel [my body]’s a little bit abandoned me and like I’m like, disconnected from it in that, like, everything I used to be able to do, I can’t do anymore. My boobs are the biggest they’ve ever been, but I don't feel sexy and I don't feel like it” (Elaine, 46).

Bodily changes could be experienced as confronting, as they forced some participants to reflect on what they represented in terms of ageing and a loss of fertility. For some, thinking about ageing was frightening or led to associating themselves with archetypes, including feeling *“like an angry old woman”* (Maeve Jasmen-O’Pause, 61). For others, it was linked to a reminder to make the most of life: *“You’re really wearing your mortality, you know, and you want to have, you know, you do want to do the best you can”* (Emily, 62). A loss of fertility was mentioned by a few participants, and with it, there was a grief or awareness of the finality of not having biological children: *“I didn’t feel old but I did feel remorse and regret that I never managed to have my own children and now it was too late”* (Sarah, 48).

Sensory sensitivities were another aspect of physical changes that were important to some, but not all, participants. Perimenopause did not appear to lessen or intensify sensory sensitivities. Instead, some participants felt that their reaction or tolerance for sensory sensitivities had changed negatively. Sensory sensitivities were more dysregulating than before: *“I feel like I could actively tune out of those things before, but I'm no longer able to do that intentionally. … Like, immediately it drives me crazy”* (Aileen, 46). They were also linked with physical discomfort, such as *“a physical response… literally from my stomach upwards, there’s a kind of shock of the noise”* (Beyonce, 46) or *“migraines or headaches from the light and, you know, just everything hurt”* (Maeve Jasmen-O’Pause, 61).

The most challenging physical symptoms were those related to participants’ sexuality, including vaginal dryness, tearing and atrophy, and libido that has *“gone off a cliff”* (Rebecca, 48). These symptoms were often positioned as impacting on partners: *“[Sex drive] becomes an issue with your partner as well. … It’s hard on them as well and because they think maybe you lose your love for them”* (Betty, 48). This was also the case for single individuals, who felt that their vaginal dryness and lowered libido would be a significant barrier to being in a relationship. These symptoms were *“depressing”* (Elaine, 46), and participants feared that they would always struggle with having sex due to pain or disinterest. Their sexuality and urogenital symptoms were considered an important target for healthcare treatment. However, several reported struggling to get their healthcare practitioners to prescribe them hormone supplements that they felt would address the issues.

### “Whatever I’m searching for perimenopause, it should be within the light of ADHD”: Navigating healthcare

Participants were passionate about the view that as women with ADHD, it was vital to receive medical support for their perimenopause, which many felt was significantly different from the neurotypical experience of perimenopause: *“For ADHD people, I just come to realise that whatever I'm searching for perimenopause, it should be within the light of ADHD, within the umbrella of ADHD, and not many people are aware of it”* (Betty, 48). However, experiences of navigating healthcare for perimenopause were mixed.

For those who had challenging experiences, several factors were reported as dissuading HCPs from identifying them as perimenopausal. Women with ADHD may present with atypical perimenopause. For example, Maeve (61) discussed not experiencing hot flashes or night sweats and her GP told her she was therefore not in perimenopause. Several participants were informed they were too young: *“I’m forty-six, constantly being told that I’m too young for what I’m experiencing to be perimenopause”* (Lynsey, 46). Additionally, multiple participants experienced their symptoms being ascribed to depression, which delayed access to HRT:

“I did go to the doctor. They said maybe I should try CBT again. The usual thing, *are you depressed? Is it the job? You're just too stressed.* And they maybe menopause was briefly mentioned, but it was dismissed very quickly.” (Rebecca, 48).

For those able to access HRT, perimenopause was marked by an effective HRT dose being prescribed: *“The threshold for me about the experience is the start of the HRT, and before and after that, that has been a dramatic effect”* (Betty, 48). HRT was reported as being effective for the cognitive, emotional, and physical changes brought by perimenopause. However, there may be some aspects of taking HRT that are not ADHD-friendly, as several women reported challenges with changes in routine, such as the oestrogen patch that requires changing twice a week and is worn for differing numbers of days. Similarly, remembering to use gels or take pills daily was at times difficult, or *“juggling”* (Elaine, 46) different types of HRT.

There was a strong belief that perimenopause care should be tailored to ADHD women, and that perimenopause and ADHD care should be integrated to fully address both. Participants felt that information about perimenopause generally may not apply to them, and the importance of having ADHD-specific psychoeducation about perimenopause available, particularly as it is challenging to find: *“To this day I still haven’t heard anybody talk about both ADHD and perimenopause/menopause together”* (Sarah, 48).

### “It’s like going through a fire that burns off all that you don’t need anymore”: Finding the positives

When reflecting on their experiences of perimenopause as women with ADHD, some participants identified aspects of their journey that brought positives. For some, it brought a new sense of self-compassion and self-understanding: *“I actually started to get gentler on myself, and well, like, okay, that explains it”* (Alison, 48). Those identified as having ADHD during perimenopause experienced the self-acceptance that came with a diagnosis. As perimenopause exacerbated ADHD traits that they had tried to *“bully”* (Elaine, 46) throughout their lives and now that they were unable due to perimenopause, they had come to accept their ADHD. Self-compassion was reinforced by perimenopause overlapping with major life events, such as a parent dying, health problems unrelated to perimenopause, or occupational stress.

An additional positive was the strengthening of their relationships with their loved ones. Several women reported how their partners and family members demonstrated love, patience, and understanding: *“I’m so lucky I have the most patient partner on the face of the earth. … It’s been very difficult with all the crying, all the moods, the snapping”* (Rebecca, 53). Friendships with other women with experience of perimenopause, ADHD or both were an important source of support and information, and several women described their passion for sharing their perimenopause journey to help others.

As described in the impact on ADHD traits subtheme, participants reported struggling to mask their ADHD traits. In contrast, there was also an intentional effort to stop masking. Masking was experienced as exhausting, and the effort needed for masking felt wasteful: *“You come to a stage where you go, I'm tired of pretending, and at this stage in my life, I'm entitled to be who I am”* (Louise, 52). This appeared to be motivated by caring less about what other people, especially strangers, think of them and a desire to let go of people pleasing.

Some women identified their perimenopause experience as a transformative journey. It offered the opportunity to find empowerment: *“It was terrifying in the beginning… I just don’t have the energy for this shit, like stop. But it has been really empowering”* (Aileen, 46). Perimenopause could bring a greater understanding of their boundaries, ADHD neurotype, and how to intentionally support themselves instead of burning out. It was also a *“new path for self-discovery”* (Miriam, 48). While perimenopause was challenging, some women with ADHD found it could bring positive change to how they viewed themselves:

“It’s like, going through a fire that burns off all that you don’t need anymore. But obviously you're walking through fire, so it’s crap. But when you come out, when you kind of settle down, and you come out the other side, you're like, built differently, and you are, I guess, a better version of yourself” (Elaine, 46).

## Discussion

The present study aimed to qualitatively understand how perimenopause impacts women with ADHD. Utilising RTA, four themes were developed. Theme 1 used two subthemes to showcase how ADHD traits can become more demanding during perimenopause, and the significant implications for mental health. Theme 2 explored how the physical symptoms of perimenopause affect relating to the body, especially sexual symptoms. Theme 3 highlighted the importance of integrated care for ADHD and perimenopause, and the supportive role of Hormone Replacement Therapy. Theme 4 suggested that while challenging, perimenopause could bring new compassion and understanding for ADHD. It is important to note that women with ADHD are at an increased risk of gynaecological disorders,^43^ which 58% of participants reported a history of, including endometriosis and fibroids. This may have contributed to experiences of perimenopause.

*“It literally just hit me like a punch in the face”: Navigating emotional and cognitive changes* identified the lived experience of how perimenopause drastically affects ADHD and mood. The subtheme *“My brain just wasn’t working properly”: The impact on ADHD traits* reflected the previous findings that a majority of women describe their ADHD as most impactful during the perimenopause stage, with pronounced cognitive difficulties,^19^ and that women with ADHD in perimenopause have higher levels of memory and concentration difficulties.^20^ Participants reported that their ADHD traits were exacerbated during this period, compared to how their ADHD previously manifested throughout their lives. It is worth noting that most participants (77%) were diagnosed during perimenopause and therefore, were relying on retrospectively reflecting on their experiences of ADHD before they had knowledge of it. It would be valuable to longitudinally investigate cognitive changes and ADHD traits in women with ADHD pre-perimenopause to post-menopause, and identify levels of change, with consideration of co-occurring conditions which may exacerbate menopause complications.

Participants in the present study reported experiencing embarrassment, frustration, and shame in relation to the cognitive changes, particularly in work settings and when changes affected their loved ones. Additionally, the impact on ADHD traits became so significant for many women in the present study that they could no longer compensate and sought a diagnosis and interventions for their ADHD. These results highlight the challenge of executive functioning for women with ADHD during perimenopause, and the importance of tailored support and understanding, and recognition of how their ADHD can be affected.

The subtheme *“This is half my life now”: The premenstrual mood, anxiety, and hormone drop* explored how the hormonal changes during perimenopause negatively affect mental health, particularly in the luteal phase. Women with ADHD are at an increased risk of Premenstrual Dysphoria Disorder (PMDD), or extreme premenstrual symptoms that impact functioning and mental health.^44^ The women in the present study identified that this worsened for them during perimenopause. Anxiety and depressive symptoms reportedly increased, in line with previous quantitative research on women with ADHD.^21,22^ For some women, the hormonal mood symptoms led to experiences of suicidal ideation. Longitudinal research has demonstrated that neurotypical women experience increased suicidal ideation during perimenopause.^45^ Given the association between ADHD and suicidal risk,^7,46^ it may be that this risk is exacerbated during perimenopause for women with ADHD. Future longitudinal research exploring perimenopause and suicidal ideation and risk for women with ADHD would be valuable. These findings also highlight the importance of considering mental health for women with ADHD in their perimenopausal care.

The theme *“I ache and I feel old”: Navigating physical changes* explored the impact of physical and sexual symptoms of perimenopause. Previous research has identified that women with ADHD experience greater levels of somatic, sexual or urogenital perimenopausal symptoms than women without ADHD.^21,22^ In contrast to qualitative research with autistic people in perimenopause,^23,24^ the women with ADHD in the present study did not identify increased sensory sensitivities. However, some reported that their reactions and tolerance to sensory experiences worsened. The most challenging physical changes were those related to sexual health. Women in perimenopause are at a greater risk of experiencing difficulties with their sexual health,^47^ and women in perimenopause experiencing greater levels of anxiety, physical and mental symptoms have significantly poorer sexual functioning.^48^ Unrelated to perimenopause, women with ADHD are more likely than neurotypical women to experience sexual functioning difficulties in desire, arousal, orgasm, satisfaction, pain, and lubrication.^49^ Therefore, perimenopausal exacerbations of executive functioning challenges and mental health difficulties may increase sexual dysfunction. Sexual well-being was an important target for intervention for the women with ADHD in the present study and was considered a core aspect of their quality of life.

The third theme, *“Whatever I’m searching for perimenopause, it should be within the light of ADHD”: Navigating healthcare*, reflected on participants’ experience of seeking and receiving healthcare for their perimenopause. Some participants identified challenging experiences and did not feel heard by their medical professionals. Women reported being dismissed if they were too young by typical standards for perimenopause, did not have the stereotypical symptoms, or their symptoms were attributed to depression. It may be that women with ADHD present with perimenopause atypically to neurotypical women. For example, Smári et al. identified that perimenopause-onset could occur at an earlier age for women with ADHD.^22^ Additionally, research on girls with ADHD has demonstrated that they are more likely to present to mental health services with mood and emotion-related symptoms.^5^ It would be valuable to explore whether psychological symptoms are the initial presentation for women with ADHD, before common physical symptoms. Perimenopause may also be underrecognised in neurodivergent people, as Benevides et al. found that only 4% of autistic individuals (*n* = 26,904) between the ages of 46-70 had medical records that included references to symptomatic menopause in their medical charts.^50^ Like those with ADHD, autistic people report significantly more perimenopause-related psychological and somatic difficulties than neurotypical people,^51^ and as such, it is likely that perimenopause is underrecognised and under-identified. It is possible it also misdiagnosed, as suggested by Theme 3 of the present study. Future research could explore the rates of menopause recognition and possible misdiagnosis among women with ADHD. Results highlight the importance of raising awareness of perimenopause in women with ADHD among healthcare professionals. Moseley et al. provide guidance on neuro-affirming care for autistic and ADHD individuals in perimenopause that offers a valuable reference point for practitioners.^35^

The final theme, *“It’s like going through a fire that burns off all that you don’t need anymore”: Finding the positives,* identified some supportive elements of perimenopause and new ways of coping. A primary feature of this theme was that perimenopause led to understanding and accepting ADHD, particularly for those who sought a diagnosis during or due to perimenopause. Perimenopause has previously been identified as a motivator for being assessed and identified as having co-occurring ADHD and autism.^52^ Other qualitative research has found the empowering effects of being diagnosed with ADHD, and that it is often accompanied by self-acceptance.^53,54^ An additional positive change was allowing themselves to unmask their ADHD, as it was too exhausting to continue to do so. A study of autistic participants in perimenopause similarly identified that, due to it being much more challenging to mask, unmasking as a result of perimenopause offered the opportunity to get to know a new version of themselves, brought a sense of relief, and a greater tendency to advocate for themselves.^27^ Alongside unmasking, perimenopause led some women with ADHD to identify new ways of supporting themselves, including recognising their limits and boundaries. Psychological interventions for women with ADHD in perimenopause could aim to capitalise on the positive transformation perimenopause can offer, and support individuals to identify new ways of coping and compensating that will support their self-acceptance, rather than trying to hide or subdue their ADHD.

### Limitations and future research

Results of the study should be considered in relation to the several limitations associated with the present study’s methodology. Firstly, participants self-reported their ADHD and that they were in perimenopause or postmenopausal. We did not confirm ADHD diagnostic status for those who reported they were formally diagnosed. Participants with self-identified ADHD were invited to participate. As perimenopause can exacerbate cognitive difficulties in neurotypical women^18,55^ and ADHD symptoms are associated with increased perimenopause symptoms in women with and without ADHD,^22^ it may be that some individuals may attribute these symptoms to ADHD. The reliability and validity of ADHD screening measures during perimenopause should be assessed, to ensure they distinguish between ADHD traits and perimenopause-related cognitive changes. It is common in neuro-affirming qualitative research to allow participants to self-report their neurotype,^54,56^ and to include individuals who are formally diagnosed or self-identify.^25,27,57^ This is especially important when conducting research with women and other marginalised individuals, as they are less likely to be diagnosed with ADHD.^4^ However, it is important to note that reliance on self-report and screening measures meant it was not possible to confirm participants were ADHD, as participants were not clinically assessed. Given the challenges with accessing diagnosis and menopause-related healthcare (as demonstrated in Theme 3), the present study opted to be as inclusive as possible. Future research may also wish to consider the benefits and costs of exclusion criteria based on validated diagnoses.

Similarly, we did not assess menopausal symptoms or status. While participants had to identify as being perimenopausal or postmenopausal to participate, we did not invite participants to self-report their menopause stage and instead relied on their last period to identify menopause stages. It is possible that last period does not necessarily align with self-identified menopause stage and future research should aim to include a measure of both.

A significant limitation of the study we did not include a screening assessment of perimenopause. For example, inclusion of screening tools such as the Meno-D^58^ or the Greene Climeractic Scale^59^ would have verified the presence of perimenopausal symptoms, along with asking participants to self-report their menopause stage. This appears to be a common limitation of qualitative research in the area of menopause,^25,60–62^ likely as it is challenging to confirm menopause and therefore, it is typically identified through self-reported symptoms.^36^ We recommend future qualitative and quantitative research consider including a measure of perimenopause, particularly given the complex relationship between ADHD traits and cognitive symptoms of perimenopause, such as brain fog. Additionally, we did not ask participants to report their history of depression and anxiety, and advise future research includes a demographic measure of previous mental health diagnoses and lifetime history of mental health symptoms.

Given the nature and aims of the present qualitative study, we did not explore whether women with ADHD had differing experiences of perimenopause if they were diagnosed before or during perimenopause, and if any factors (e.g., medication use) influenced their experience of perimenopause. This would be a valuable avenue for future research. A key target for research is understanding why ADHD traits are exacerbated during perimenopause.^43^ As such, identifying if an earlier diagnosis and treatment can reduce the impact of perimenopause could provide further insight into the relationship. Similarly, the present study did not explore differences in experiences if an individual was in perimenopause due to natural or chemical means. Women with ADHD appear to be sensitive to the side effects of hormonal contraception.^8^ Therefore, it would be helpful to explore how women with ADHD experience chemically-induced perimenopause. No participants had experienced surgical menopause. While participants who had experienced surgical menopause were welcome to participate to be as inclusive as possible, given the severity and potential trauma associated with it, future research might consider investigating this as a unique form of menopause for neurodivergent women.

All participants were cisgender women, although study advertisements and materials utilised gender-neutral language. Additionally, all participants were heterosexual or in an opposite-sex relationship. Research has suggested that women in LGBTQ+ relationships may experience fewer sexual functioning challenges during perimenopause, possibly due to improved communication about sexual changes and both partners having decreased desire simultaneously.^63^ It may be that the emphasis on sexual functioning difficulties in the present study relates to being in an opposite-sex partnership. It would be valuable to explore the experiences of perimenopause for non-binary and trans individuals with ADHD, and women and genderfluid people with ADHD in LGBTQ+ relationships.

## Conclusion

In this qualitative study, we aimed to explore the lived experience of perimenopause for women with ADHD. Results highlight the significant impact of perimenopause as it exacerbates ADHD traits and mood symptoms during hormonal fluctuations, the negative effects of physical symptoms on quality of life, and the challenges of navigating healthcare for perimenopause as women with ADHD. Findings also highlighted that some women had a positive outlook and felt that perimenopause brought a welcome change to their lives. Overall, the study highlights how challenging perimenopause can be for women with ADHD, and the importance of tailored support and interventions.

## Supplemental material

Supplemental material - “Hormones rule me”: A qualitative exploration of the impact of perimenopause on women with ADHD Supplemental material for “Hormones rule me”: A qualitative exploration of the impact of perimenopause on women with ADHD by Christina Kini-Seery, Samantha Trevaskis, Ken Kilbride, Margo Wrigley, & Jessica Bramham in Women’s Health

## Data Availability

Data is available upon reasonable request from the corresponding author.*

## References

[1] American Psychiatric Association . Diagnostic and statistical manual of mental disorders: DSM-5-TR^TM^. American Psychiatric Publishing, Inc., 2024.

[2] Song P Zha M Yang Q , et al. The prevalence of adult attention-deficit hyperactivity disorder: A global systematic review and meta-analysis. J Glob Health 2021; 11: 04009. 10.7189/jogh.11.04009 33692893 PMC7916320

[3] ten Have M Tuithof M van Dorsselaer S , et al. Prevalence and trends of common mental disorders from 2007-2009 to 2019-2022: results from the Netherlands Mental Health Survey and Incidence Studies (NEMESIS), including comparison of prevalence rates before vs. during the COVID-19 pandemic. World Psychiatry 2023; 22: 275–285. 10.1002/wps.21087 37159351 PMC10168151

[4] Martin J . Why are females less likely to be diagnosed with ADHD in childhood than males? The Lancet Psychiatry 2024; 11: 303–310. 10.1016/S2215-0366(24)00010-5 38340761

[5] Martin J Langley K Cooper M , et al. Sex differences in attention-deficit hyperactivity disorder diagnosis and clinical care: a national study of population healthcare records in Wales. J Child Psychol Psychiatry 2024; 65: 1648–1658. 10.1111/jcpp.13987 38864317

[6] Skoglund C Sundström Poromaa I Leksell D , et al. Time after time: failure to identify and support females with ADHD – a Swedish population register study. Journal of Child Psychology and Psychiatry 2023; 65(6): 832–844. 10.1111/jcpp.13920 38016697

[7] Fuller-Thomson E Rivière RN Carrique L , et al. The Dark Side of ADHD: Factors Associated With Suicide Attempts Among Those With ADHD in a National Representative Canadian Sample. Archives of Suicide Research 2022; 26: 1122–1140. 10.1080/13811118.2020.1856258 33345733

[8] Lundin C Wikman A Wikman P , et al. Hormonal Contraceptive Use and Risk of Depression Among Young Women With Attention-Deficit/Hyperactivity Disorder. Journal of the American Academy of Child & Adolescent Psychiatry 2023; 62: 665–674. 10.1016/j.jaac.2022.07.847 36332846

[9] Hartman CA Larsson H Vos M , et al. Anxiety, mood, and substance use disorders in adult men and women with and without attention-deficit/hyperactivity disorder: A substantive and methodological overview. Neuroscience & Biobehavioral Reviews 2023; 151: 105209. 10.1016/j.neubiorev.2023.105209 37149075

[10] Hinshaw SP Nguyen PT O’Grady SM , et al. Annual Research Review: Attention-deficit/hyperactivity disorder in girls and women: underrepresentation, longitudinal processes, and key directions. Journal of Child Psychology and Psychiatry 2022; 63: 484–496. 10.1111/jcpp.13480 34231220

[11] Osianlis E Thomas EHX Jenkins LM , et al. ADHD and Sex Hormones in Females: A Systematic Review. J Atten Disord 2025; 29: 706–723. 10.1177/10870547251332319 40251875 PMC12145478

[12] Findeis H Strauß M . The effects of psychostimulants in menstruating women with ADHD – A gender health gap in ADHD treatment? Progress in Neuro-Psychopharmacology and Biological Psychiatry 2025; 137: 111261. 10.1016/j.pnpbp.2025.111261 39837362

[13] Dorani F Bijlenga D Beekman ATF , et al. Prevalence of hormone-related mood disorder symptoms in women with ADHD. Journal of Psychiatric Research 2021; 133: 10–15. 10.1016/j.jpsychires.2020.12.005 33302160

[14] Kooij JS . Hormonal sensitivity of mood symptoms in women with ADHD across the lifespan. European Psychiatry 2023; 66: S23.

[15] Santoro N . Perimenopause: From Research to Practice. J Womens Health (Larchmt) 2016; 25: 332–339. 10.1089/jwh.2015.5556 26653408 PMC4834516

[16] Wynchank D Jong MD Kooij SJJS . Practical tools for female-specific ADHD: The impact of hormonal fluctuations in clinical practice and from the literature. European Psychiatry 2026; 69: e1. 10.1192/j.eurpsy.2025.10120 PMC12816923 41115846

[17] Roberts B Eisenlohr-Moul T Martel MM . Reproductive steroids and ADHD symptoms across the menstrual cycle. Psychoneuroendocrinology 2018; 88: 105–114. 10.1016/j.psyneuen.2017.11.015 29197795 PMC5803442

[18] Metcalf CA Duffy KA Page CE , et al. Cognitive Problems in Perimenopause: A Review of Recent Evidence. Curr Psychiatry Rep 2023; 25: 501–511. 10.1007/s11920-023-01447-3 37755656 PMC10842974

[19] Wasserstein J Stefanatos GA Solanto MV . 2 Perimenopause, Menopause and ADHD. Journal of the International Neuropsychological Society 2023; 29: 881. 10.1017/s1355617723010846

[20] Chapman L Gupta K Hunter MS , et al. Examining the Link Between ADHD Symptoms and Menopausal Experiences. J Atten Disord 2025; 29(14): 1263–1277. 10.1177/10870547251355006 40738484 PMC12569137

[21] Boyd C . Adults with ADHD in Ireland: Services, supports, and needs. University College, 2024.

[22] Smári UJ Valdimarsdottir UA Wynchank D , et al. Perimenopausal symptoms in women with and without ADHD: A population-based cohort study. European Psychiatry 2025; 68: e133. 10.1192/j.eurpsy.2025.10101 40903825 PMC12538516

[23] Moseley RL Druce T Turner-Cobb JM . When my autism broke’: A qualitative study spotlighting autistic voices on menopause. Autism 2020; 24: 1423–1437. 10.1177/1362361319901184 32003226 PMC7376624

[24] Moseley RL Druce T Turner-Cobb JM . Autism research is ‘all about the blokes and the kids’: Autistic women breaking the silence on menopause. British Journal of Health Psychology 2021; 26: 709–726. 10.1111/bjhp.12477 32996665

[25] Brady MJ Jenkins CA Gamble-Turner JM , et al. “A perfect storm”: Autistic experiences of menopause and midlife. Autism 2024; 28: 1405–1418. 10.1177/13623613241244548 38622794 PMC11135000

[26] Kessler RC Adler L Ames M , et al. The World Health Organization adult ADHD self-report scale (ASRS): a short screening scale for use in the general population. Psychological Medicine 2005; 35: 245–256. 10.1017/s0033291704002892 15841682

[27] Jenkins CA Moseley RL Matthews RJ , et al. “Struggling for Years”: An international survey on Autistic experiences of menopause. Neurodiversity 2024; 2: 27546330241299366. 10.1177/27546330241299366

[28] Tong A Sainsbury P Craig J . Consolidated criteria for reporting qualitative research (COREQ): a 32-item checklist for interviews and focus groups. Int J Qual Health Care 2007; 19: 349–357. 10.1093/intqhc/mzm042 17872937

[29] Kessler RC Adler LA Gruber MJ , et al. Validity of the World Health Organization Adult ADHD Self-Report Scale (ASRS) Screener in a representative sample of health plan members. International Journal of Methods in Psychiatric Research 2007; 16: 52–65. 10.1002/mpr.208 17623385 PMC2044504

[30] Chapman R Botha M . Neurodivergence-informed therapy. Developmental Medicine & Child Neurology 2023; 65: 310–317. 10.1111/dmcn.15384 36082483

[31] To saturate or not to saturate? . Questioning data saturation as a useful concept for thematic analysis and sample-size rationales. Qualitative Research in Sport, Exercise and Health. 10.1080/2159676X.2019.1704846 (accessed 18 March 2026).

[32] Husted M Dowrick A Porter R , et al. Imposter Participants in Synchronous Qualitative Research: A Systematic Scoping Review. International Journal of Qualitative Methods 2025; 24: 16094069251342542. 10.1177/16094069251342542

[33] Lough Dennell BL Dowrick A Evered JA , et al. Do You Know Who You’re Talking To? Methodological Reflections on Maintaining Inclusivity and Research Integrity When Responding to Inauthentic Encounters in Online Qualitative Research. International Journal of Qualitative Methods 2025; 24: 16094069251384112. 10.1177/16094069251384112

[34] Brown TE . ADD/ADHD and Impaired Executive Function in Clinical Practice. Curr Psychiatry Rep 2008; 10: 407–411. 10.1007/s11920-008-0065-7 18803914

[35] Moseley RL Gamble-Turner JM Kim E , et al. Autism, ADHD and the menopause. Post Reproductive Health 2026; 1–11. 10.1080/20533691.2026.263122242439012

[36] National Institute for Health and Care Excellence . Menopause: Identification and management, 2024. https://www.nice.org.uk/guidance/ng23 31940155

[37] Braun V Clarke V . Thematic Analysis: A Practical Guide. Sage, 2021.

[38] Houston S . Beyond Social Constructionism: Critical Realism and Social Work. The British Journal of Social Work 2001; 31: 845–861. 10.1093/bjsw/31.6.845

[39] Pilgrim D . Some implications of critical realism for mental health research. Soc Theory Health 2014; 12: 1–21. 10.1057/sth.2013.17

[40] Lumivero. NVivo.

[41] Seery C Leonard-Curtin A Naismith L , et al. The understanding and managing adult ADHD programme: A qualitative evaluation of online psychoeducation with acceptance and commitment therapy for adults with ADHD. Journal of Contextual Behavioral Science 2023; 29: 254–263. 10.1016/j.jcbs.2023.08.005

[42] Seery C Leonard-Curtin A Naismith L , et al. Feasibility of the Understanding and Managing Adult ADHD Programme: open-access online group psychoeducation and acceptance and commitment therapy for adults with attention-deficit hyperactivity disorder. BJPsych Open 2024; 10: e163. 10.1192/bjo.2024.743 39324244 PMC11457231

[43] Kooij JJS de Jong M Agnew-Blais J , et al. Research advances and future directions in female ADHD: the lifelong interplay of hormonal fluctuations with mood, cognition, and disease. Front Glob Womens Health 2025; 6: 1613628. 10.3389/fgwh.2025.1613628 40692967 PMC12277363

[44] Broughton T Lambert E Wertz J , et al. Increased risk of provisional premenstrual dysphoric disorder (PMDD) among females with attention-deficit hyperactivity disorder (ADHD): cross-sectional survey study. The British Journal of Psychiatry 2025; 226: 410–417. 10.1192/bjp.2025.104 40528384 PMC7617793

[45] Nakanishi M Endo K Yamasaki S , et al. Association between menopause and suicidal ideation in mothers of adolescents: A longitudinal study using data from a population-based cohort. Journal of Affective Disorders 2023; 340: 529–534. 10.1016/j.jad.2023.08.055 37573891

[46] Jeng J-S Huang H-H Chang W-H , et al. Longitudinal study on all-cause and suicide mortality among individuals with attention deficit hyperactivity disorder. Eur Child Adolesc Psychiatry 2025; 34: 1229–1238. 10.1007/s00787-024-02511-w 38916769

[47] Wang Y Islam RM Bond M , et al. Sexual dysfunction in women at midlife: a cross-sectional study of data from the Australian Women’s Midlife Years study. The Lancet Obstetrics, Gynaecology, & Women’s Health 2025; 1: e198–e208. 10.1016/j.lanogw.2025.100024

[48] Saleh SA Almadani N Mahfouz R , et al. Exploring the Intersection of Depression, Anxiety, and Sexual Health in Perimenopausal Women. International Journal of Women’s Health 2024; 16: 1315–1327. 10.2147/IJWH.S464129 PMC11298183 39100112

[49] Amani Jabalkandi S Raisi F Shahrivar Z , et al. A study on sexual functioning in adults with attention-deficit/hyperactivity disorder. Perspectives in Psychiatric Care 2020; 56: 642–648. 10.1111/ppc.12480 32043624

[50] Benevides TW Cook B Klinger LG , et al. Brief Report: Under-Identification of Symptomatic Menopause in Publicly-Insured Autistic People. J Autism Dev Disord 2024; 10.1007/s10803-024-06516-x PMC12938728 39210156

[51] Charlton RA Happé FG Shand AJ , et al. Self-Reported Psychological, Somatic, and Vasomotor Symptoms at Different Stages of the Menopause for Autistic and Non-autistic People. Journal of Women’s Health 2025; 34: 622–634. 10.1089/jwh.2024.0784 39960820

[52] Craddock E . Being a Woman Is 100% Significant to My Experiences of Attention Deficit Hyperactivity Disorder and Autism: Exploring the Gendered Implications of an Adulthood Combined Autism and Attention Deficit Hyperactivity Disorder Diagnosis. Qual Health Res 2024; 34: 1442–1455. 10.1177/10497323241253412 39025117 PMC11580322

[53] Babinski DE Libsack EJ . Adult Diagnosis of ADHD in Women: A Mixed Methods Investigation. J Atten Disord 2025; 29: 207–219. 10.1177/10870547241297897 39588653 PMC11694561

[54] Morgan J . Exploring women’s experiences of diagnosis of ADHD in adulthood: a qualitative study. Advances in Mental Health 2024; 22: 575–589. 10.1080/18387357.2023.2268756

[55] Smith W Cooney SM Naraindas AM . The influence of menopause symptoms on workplace mental health among Irish women: A preliminary study. Comprehensive Psychoneuroendocrinology 2025; 24: 100324. 10.1016/j.cpnec.2025.100324 41211164 PMC12595140

[56] Bürger I Erlandsson K Borneskog C . Perceived associations between the menstrual cycle and Attention Deficit Hyperactivity Disorder (ADHD): A qualitative interview study exploring lived experiences. Sexual & Reproductive Healthcare 2024; 40: 100975. 10.1016/j.srhc.2024.100975 38678676

[57] Beaton DM Sirois F Milne E . Experiences of criticism in adults with ADHD: A qualitative study. PLOS ONE 2022; 17: e0263366. 10.1371/journal.pone.0263366 35180241 PMC8856522

[58] Kulkarni J Gavrilidis E Hudaib A-R , et al. Development and validation of a new rating scale for perimenopausal depression—the Meno-D. Transl Psychiatry 2018; 8: 123. 10.1038/s41398-018-0172-0 29955034 PMC6023883

[59] Greene JG . Constructing a standard climacteric scale. Maturitas 1998; 29: 25–31. 10.1016/s0378-5122(98)00025-5 9643514

[60] Refaei M Mardanpour S Masoumi SZ , et al. Women’s experiences in the transition to menopause: a qualitative research. BMC Womens Health 2022; 22: 53. 10.1186/s12905-022-01633-0 35219295 PMC8882304

[61] Matina SS Mendenhall E Cohen E . Women´s experiences of menopause: A qualitative study among women in Soweto, South Africa. Global Public Health 2024; 19: 2326013. 10.1080/17441692.2024.2326013 38497205

[62] Hendriks O McIntyre JC Rose AK , et al. The mental health challenges, especially suicidality, experienced by women during perimenopause and menopause: A qualitative study. Womens Health (Lond Engl) 2025; 21: 17455057251338941. 10.1177/17455057251338941 40626330 PMC12246672

[63] Sobel T Derakshani D Vencill JA . Menopause experiences in sexual minority women and non-binary people. Maturitas 2024; 185: 108007. 10.1016/j.maturitas.2024.108007 38677174

